# School absenteeism data for surveillance purposes: a proxy for acute respiratory infection rates

**DOI:** 10.1186/s12889-026-27014-y

**Published:** 2026-03-17

**Authors:** C. M. de Korne, M. Hooiveld, A. J. van Hoek, P. C.J.L Bruijning-Verhagen

**Affiliations:** 1 Julius Center for Health Sciences and Primary Care, UMC Utrecht, Utrecht, The Netherlands; 2 Nivel, Utrecht, the Netherlands; 3 Centre for Infectious Disease Control, National Institute for Public Health and the Environment, Bilthoven, The Netherlands

**Keywords:** School absenteeism, Respiratory infections, Syndromic surveillance, School outbreaks

## Abstract

**Background:**

School absenteeism data have potential as real-time public health surveillance tool to monitor infectious diseases. This study evaluates the validity and usefulness of school absenteeism data for application in acute respiratory illness (ARI) surveillance.

**Methods:**

Five Dutch primary schools participated in a study (2022/23) to determine reasons for absence using illness surveys. Results were clustered into ARI and non-ARI illness. Spatiotemporal clustering of illness-related absence episodes within the five schools was assessed. A second dataset on absenteeism from 69 primary schools was used to examine longer term absenteeism trends (school years 2017/18, 2018/19, 2022/23) and school-level characteristics associated with absenteeism rates. We also assessed correlations between these absenteeism rates and the corresponding ARI-related primary care visit rates for children aged 5–9.

**Results:**

In the five primary schools, ARI accounted for two-thirds of illness-related absenteeism and showed clear spatiotemporal clustering by school and class. Across the 69 primary schools, absenteeism rates exhibited a strong seasonal pattern and higher rates were significantly associated with lower neighbourhood socio-economic status. Post-pandemic absenteeism exceeded pre-pandemic levels by twofold with a mean rate of 2.2 absent days/100 school days in 2022/23 (equivalent to ~ 5 absent days per child/school year). Time trends in absenteeism rates correlated strongly with ARI primary care visit rates, with correlation coefficients > 0.8.

**Conclusion:**

School absenteeism is strongly linked to respiratory illness and children’s primary care visits for ARI. Absenteeism patterns reflect key characteristics of ARI trends including spatiotemporal clustering and seasonality. School absenteeism data is therefore suitable for ARI surveillance.

**Supplementary Information:**

The online version contains supplementary material available at 10.1186/s12889-026-27014-y.

## Introduction

 School-aged children are known the be important drivers of respiratory pathogen transmission due to their frequent and close interactions in educational settings and elsewhere [1, 2]. Therefore, monitoring acute respiratory illness (ARI) rates in school-aged children could be a valuable component of syndromic community surveillance. Traditional ARI surveillance relies on cases reported by healthcare facilities, with or without pathogen confirmation [3]. This method overlooks the vast majority of milder ARI cases that do not seek medical attention and has limited granularity [4]. Participatory surveillance systems such as ‘Infectieradar’, which rely on voluntary symptom reporting, capture milder ARI cases, but underrepresent school-aged children [5]. Active surveillance within schools could address this gap and provide detailed, location-specific data. However, this approach is intrusive, time-consuming, and resource-intensive, making it impractical for large-scale monitoring [6].

School absenteeism has emerged as a promising proxy to monitor ARI rates in school-aged children. It provides a non-intrusive, real-time data source with high temporal and spatial resolution [7, 8]. However, disadvantages include the lack of specificity regarding the cause of absence, missing data during weekends and holidays and inconsistencies in reporting practices [9, 10]. Recent reviews have concluded that there is a correlation between school absenteeism rates and influenza activity in the community, but the results varied widely across studies [11, 12]. This variability is likely due to differences in the number of schools involved (ranging from a few to several thousand), the age groups studied (primary schools versus secondary schools), study periods (specific outbreaks like the 2009 H1N1 pandemic versus multiple influenza seasons), types of absenteeism data used (all-cause versus illness-related absences) and the nature of community illness data used as a reference (e.g. primary care consultation rates or hospitalizations from children versus all age groups). Consequently, it remains uncertain to what extent illness-related absenteeism data reflect ARI rates in school-aged children.

In the Netherlands, all schools are mandated to record student absenteeism daily in a digital administrative system, in accordance with the Compulsory Education Act of 1969. Schools are required to classify each absence as either authorized or unauthorized, with authorized absences including specific exceptions, such as family events (e.g., weddings, funerals) or illnesses reported by parents within two days of the child’s absence. This categorization is overseen by the National Education Inspection to ensure compliance [13]. In primary schools, absence entries often include a free-text reason provided by the school, however the level of detail varies, as schools have considerable flexibility in how these reasons are recorded.

This study aimed to validate the use of illness-related school absenteeism rates as a proxy for ARI rates among primary school-aged children in the Netherlands. Our validation study included several complementary analyses that jointly provide insight in the value of school-absenteeism data for ARI surveillance and public health strategies.

## Methods

In the Netherlands, primary education consists of eight grades, typically attended by children aged 4 to 12. To validate illness-related school absenteeism rates as a proxy for ARI rates, we conducted a stepwise analysis using four complementary datasets: illness survey data on reasons for absence and class-level absenteeism data from five primary schools for school-year 2022/23, school-level absenteeism data for school-years 2017/18, 2018/19, 2022/23 from 69 schools across the Netherlands and national ARI-related primary care visit data for the same years. First, we analysed the survey data to determine reasons for absenteeism, focusing on the contribution of ARI. Next, we examined temporal trends in absenteeism across and within schools and evaluated associations with school characteristics. Finally, we assessed the temporal correlation between absenteeism rates and ARI primary care visit rates.

### Data sources

#### Illness survey data

To gain insight into the type of illnesses contributing to absenteeism, an illness survey was conducted during a seven-week period (Nov-Dec 2023) in five schools in the province of Limburg participating in the pilot study on air-cleaning devices [14]. Parents of absent students were invited to complete an anonymous online survey on the first day of absence, reporting the reason for absence and symptoms, and presence of ARI symptoms in other members of the household. Reported symptoms were categorized into ARI, gastrointestinal illness (GI) where possible, and cases with non-specific symptoms where assigned to the other/unknown category. ARI and GI cases were further classified as likely school-related or not, based on whether similar symptoms had been reported days earlier among any of the child’s household members. Definitions for both categorizations are provided in Supplement 1.

#### Class-level absenteeism data

A class-level absenteeism dataset was obtained from the same five schools, who provided an export from their administrative systems. The export covered the full 2022/23 school-year (and the study period, which was not used in the present analyses). This dataset included unique absence episodes, each with a start and end date and a recorded reason: unauthorized or authorized, with the latter further divided into illness-related or other reasons. We included all illness-related absences in the analysis. Both illness absence episodes and the number of registered students were recorded per school, grade, and class. Grade 1 data were excluded because of variable class sizes throughout the year, and only end-of-year group sizes were available.

#### School-level absenteeism data

Data from the school administrative system ParnasSys™ were used to obtain a school-level absenteeism dataset from 69 Dutch primary schools across the Netherlands. All absences were routinely recorded in the administrative system by the schools. Records were subsequently extracted, anonymized and aggregated by The Implementation Group (TIG) with permission from the school-boards. The dataset contained daily absence counts per school and the total number of enrolled students. The geographical distribution of the schools is depicted in Supplementary Figure S1A. Next, the absenteeism data were linked via a unique school identifier (BRIN) to open-access school-level data from the Dutch Education Executive Agency (in Dutch abbreviated as ‘DUO’). Details on the included schools, the linked DUO variables, and their national representativeness are provided in Supplement 2. For this validation study, we focused on non-pandemic data, using two pre-pandemic school years (2017/18 and 2018/19) and one post-pandemic school year (2022/23). Data from the pandemic years were excluded because school absenteeism was heavily influenced by mitigation measures such as quarantine and remote education and reporting was inconsistent. Absences classified as illness-related were included in the analysis. These are authorized absences reported as due to illness. Unlike in some neighboring countries, Dutch schools rely on parental reporting of sickness and do not require a doctor’s note. In a sensitivity analysis, we included all authorized absences (irrespective of documentation on illness). These definitions are explained in Supplement 3.

#### ARI primary care visits

The Netherlands Institute for Health Services Research (Nivel) has a Primary Care Database which routinely records data from GPs to monitor health and healthcare use in a representative sample of the Dutch population [15, 16]. For this study, the database was used to determine GP visit rates for ARI during periods covered by the absenteeism data. The dataset contained the weekly number of GP consultations from over 400 GP practices across the Netherlands, stratified by 5-year age groups and by municipal health service (in Dutch abbreviated as ‘GGD’) regions, and classified using the International Classification of Primary Care (ICPC) codes. The geographical distribution of participating GP practices is depicted in Supplementary Figure S2. ARI was defined using the following ICPC codes: R74 (acute upper respiratory infection), R75 (acute/chronic rhinosinusitis), R77 (acute laryngitis/tracheitis), R78 (acute bronchitis/bronchiolitis), R80 (influenza), or R81 (pneumonia). As the study schools were located in the GGD regions of Amsterdam, Hollands-Midden and Limburg, we selected these three regions from the Nivel dataset to match the geographical coverage of the absenteeism data. Because ARI primary care data were available only in 5-year age groups, the 5-9-year age group was selected as the closest available approximation to primary school-aged children (4–12 years). Weekly GP-visit rates for ARI were expressed as the number of persons with ARI-related visits per 10,000 persons in this age group.

### Statistical analysis

From the illness survey data we determined the distribution of reported symptoms among students reporting absent and the proportion of illnesses categorized as ARI, GI, or other/unknown across different age groups. These were grouped by school grades 1–2 (ages 4–6), grades 3–5 (ages 7–9), and grades 6–8 (ages 10–12). Differences in symptoms prevalence between age groups were assessed using a Chi-square test. From the class-level absenteeism dataset from the same schools we quantified the weekly incidence of illness-related absence episodes, defined as the number of new absenteeism episodes divided by the total student school days at risk, with students present the previous day considered eligible to become newly absent. Spatiotemporal clustering of absenteeism was explored visually by plotting IRs for each class over time, using circles sized proportionally to their value.

The school-level absenteeism dataset from 69 schools was used to estimate weekly absenteeism rates for the school-years 2017/18, 2018/19 and 2022/23. We applied a multilevel generalized linear mixed-effects model (GLMM) with a negative binomial distribution, as absenteeism data are counts. The weekly number of absent days per school was modelled with the logarithm of student school days (the number of days students were expected to attend; e.g. 5 days multiplied by the number of enrolled students in weeks without holidays) as an offset term, with no overall intercept, school year as a covariate, and random effects for school and week:

$$\mathrm{log}\left(E\left[{Y}_{i,t}\right]\right)=\mathrm{log}\left({Schooldays}_{i,t}\right)+{\beta}_{Schoolyear}+{u}_{i}+{v}_{t}$$

This yields a weekly absenteeism rate per school year which was expressed as absent days/100 school days. A similar model structure was used to estimate weekly absenteeism rates by school period (i.e. the intervals between holidays), with school period included as a fixed effect.

To estimate the cumulative number of absent days per child over the school year, we also used a GLMM in which the mean total annual number of absent days per school was modelled with the logarithm of enrolled students as an offset term, with no overall intercept, school year as a covariate, and a random effect for school:

$$\mathrm{log}\left(E\left[{Y}_{i}\right]\right)=\mathrm{log}\left({Enrolledstudents}_{i}\right)+{\beta}_{Schoolyear}+{u}_{i}$$

This yields the mean number of absent days per student per school year.

Next, to examine determinants of absenteeism, this model was extended with the following school-level variables as fixed effects: number of children enrolled, school performance, socio-economic status, urbanity, percentage of children with a non-Dutch cultural background, and average commuting distance of students. Definitions of these variables are provided in Supplement 2. Differences between pre-pandemic (2017/18, 2018/19) and post-pandemic (2022/23) school years were assessed by calculating the number of absent days per child separately per year.

The correlation between illness-related absenteeism and ARI primary care visit rates was assessed based on Spearman’s rank correlation to capture monotonic trends, and Pearson correlation was applied to evaluate linear associations. To assess temporal alignment, we conducted cross-correlation analysis, examining time lags from − 5 to + 5 weeks, a range chosen to reflect plausible lead-lag relationships between school absenteeism and GP consultations. Prior to cross-correlation, both time series were normalized to ensure comparability and focus on relative changes rather than absolute levels. To determine whether the relationship between absenteeism and primary care visits remained consistent over time, we calculated the ‘absenteeism-to-GP-visits’ ratio for each school year during the peak season (Autumn to Spring break). This ratio was defined as the illness-related absenteeism rate (weekly absent days per 50.000 school days) divided by the ARI primary care visit rates (weekly visits per 10.000 persons).

#### Analysis software

All data processing, visualization, and most statistical analyses were performed using Python (v3.11.5). GLMMs were fitted in R (v4.1.3) using the glmmTMB package (v1.1.9).

## Results

### Reasons for absenteeism

**Fig. 1:**
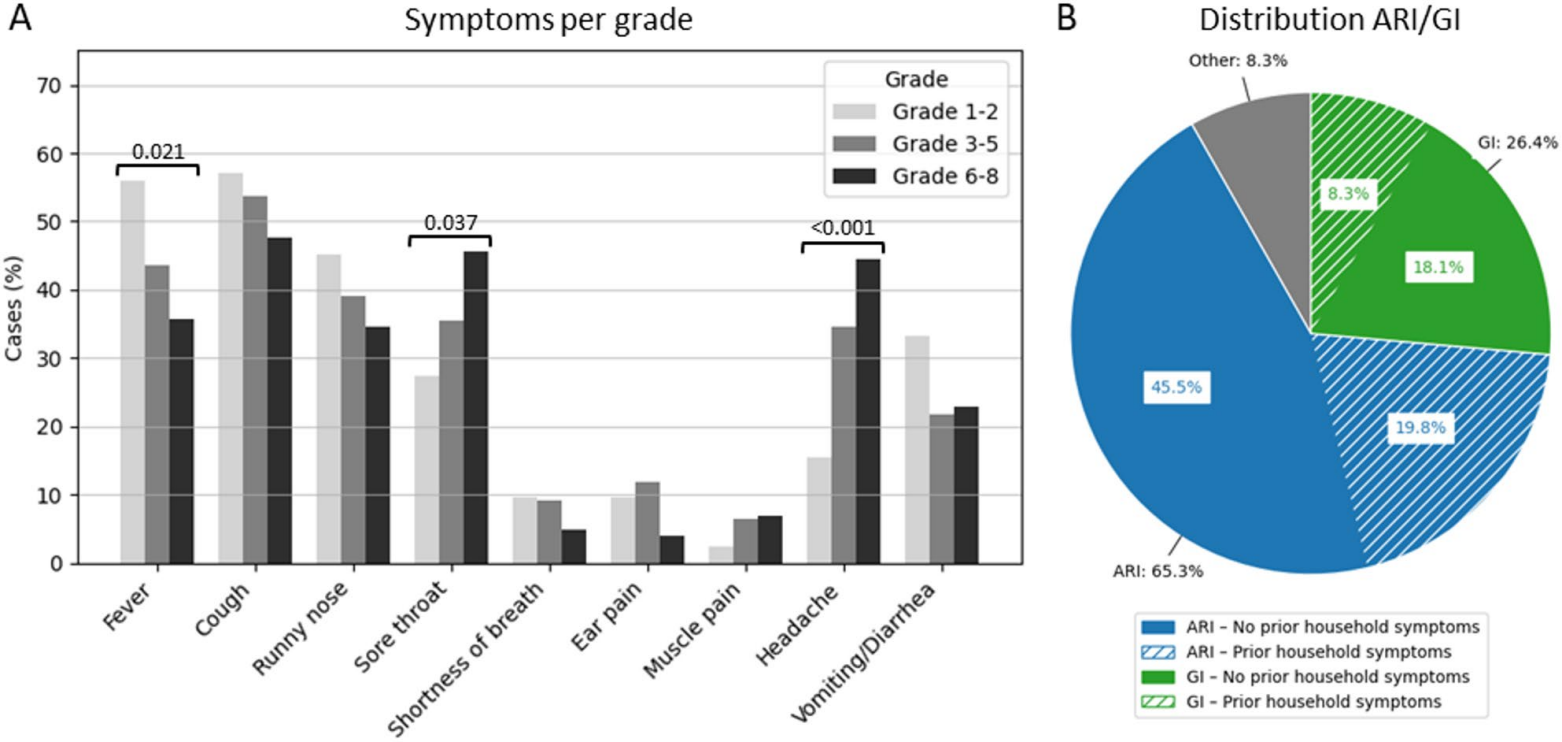
**A** Distribution of symptoms reported in illness surveys by grade groups (1–2, 3–5, 6–8), shown as the percentage of cases with each symptom. Differences in symptom distribution across grade groups were assessed using a Chi-square test. **B** The proportion of illnesses categorized as attributable to acute respiratory infections (ARI), gastrointestinal illness (GI) or other/unknown. The ARI and GI episodes are further subdivided into cases where symptoms were already present in the household and cases where the child was the index case

In November and December 2023, 371 surveys were distributed among parents of children reported absent due to illness, achieving a completion rate of 81.7% across the five participating schools. Cough was the most commonly reported symptom. Symptom occurrence varied by age group, with older children (grades 6–8) more frequently reporting headache and sore throat, and younger children (grades 1–3) more often reporting fever (resp. p-values: 0.021, 0.037, < 0.001; Chi-square test; Fig. 1A). The majority (65.3%) of the illness absenteeism was classified as ARI, while 26.4% was classified as attributable to GI (Fig. 1B). In 30.3% of the ARI episodes and 31.6% of the GI episodes, symptoms had started earlier in at least one other household member. For the remaining episodes, the child was the index case who likely contracted the illness outside the household, possibly at school (Fig. 1B).

### Clustering of absences

**Fig. 2:**
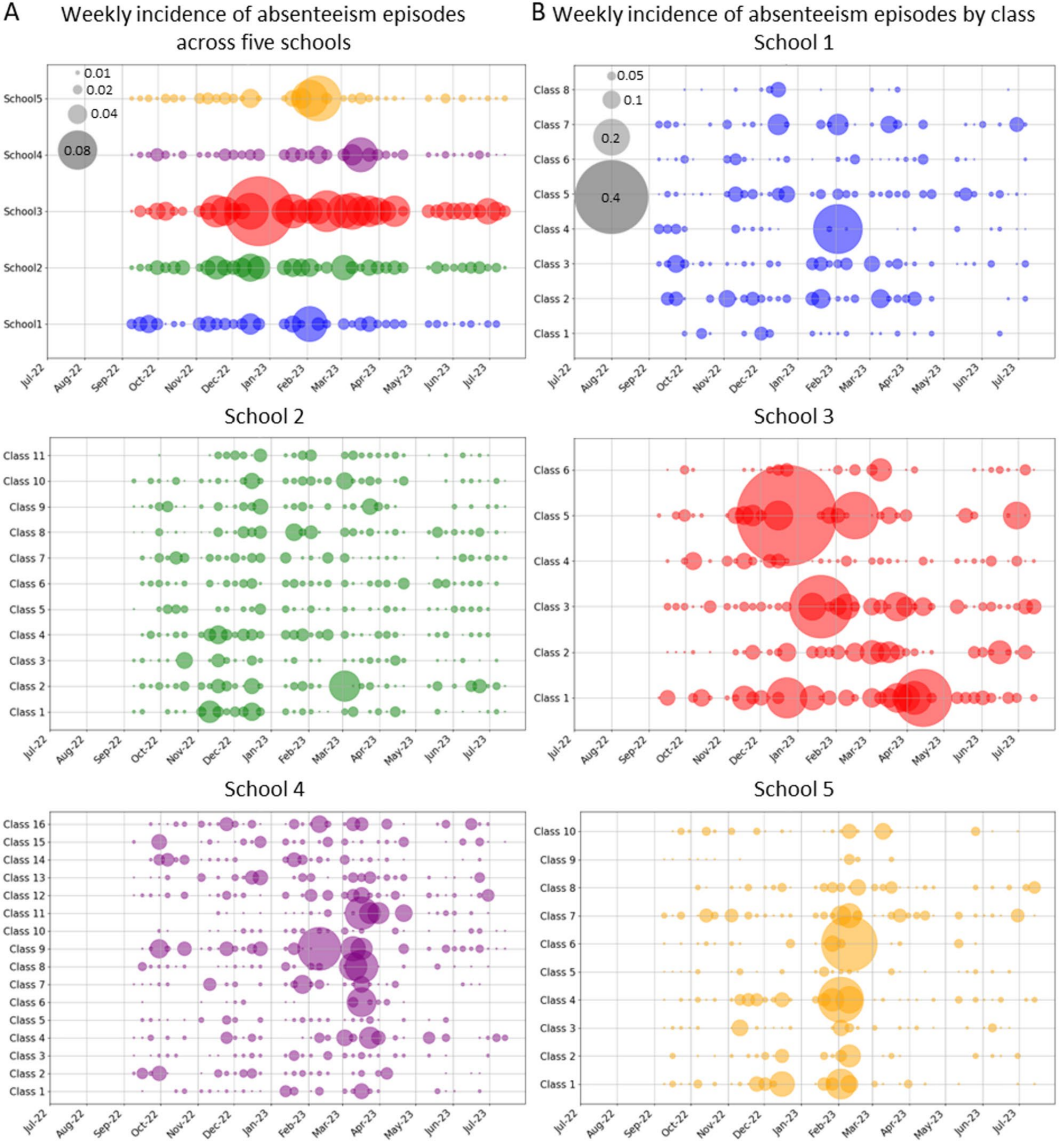
Weekly absenteeism rates over time per school (**A**) and per class (**B**, School 1–5). The size of the circles represents the magnitude of the incidence rate, visualizing the clustering of absenteeism

Visual inspection of the weekly incidence of illness-related absence episodes by school and over time showed clustering within schools and particularly within classes. The extent and timing of clustering varied across schools and classes, with some exhibiting sharp peaks in new episodes during specific weeks, most notably in School 3 (Fig. 2AB).

### Temporal trends in illness-related absenteeism rates

**Fig. 3:**
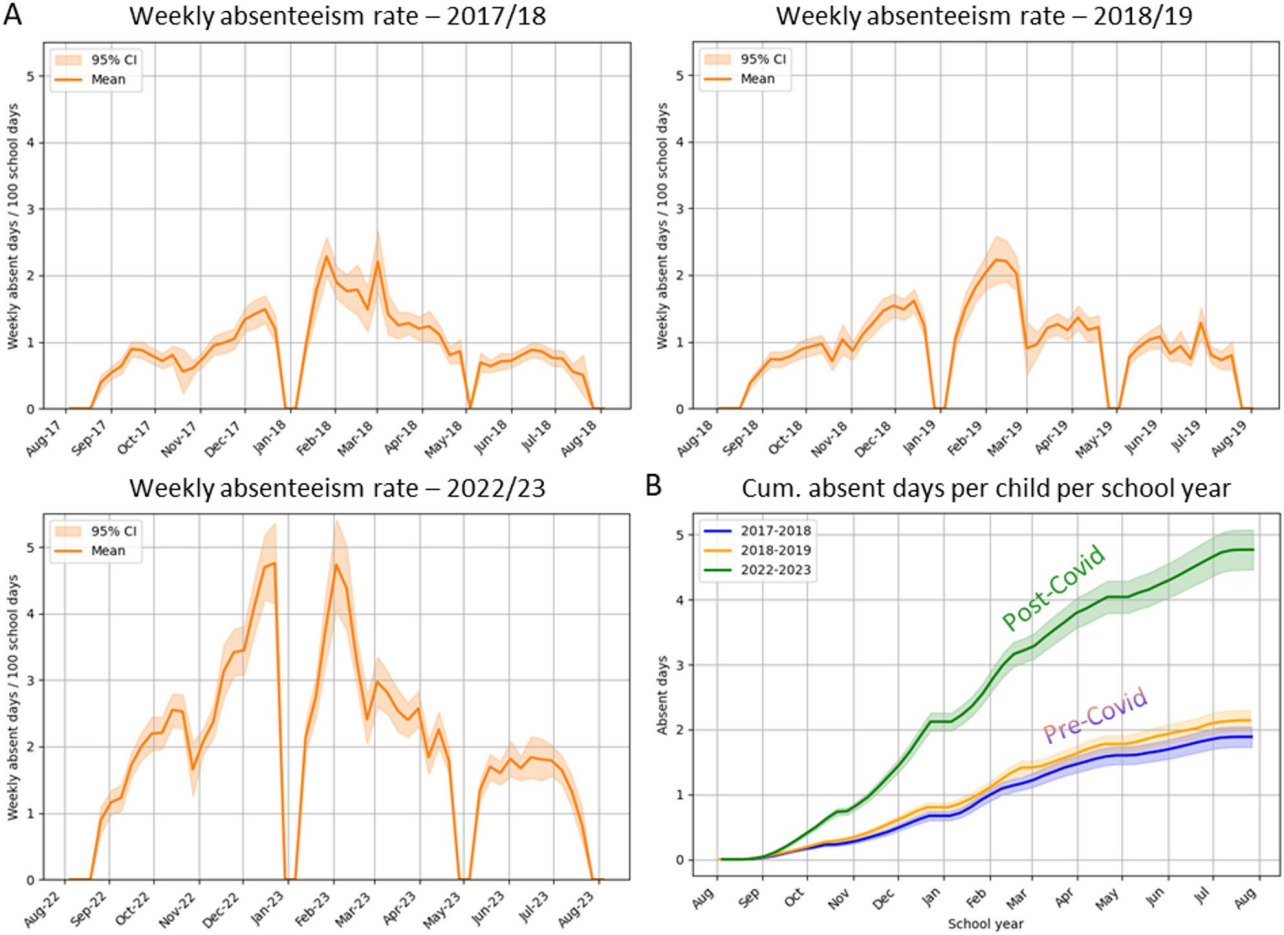
**A** Mean weekly school-level illness-related absenteeism rates for the 2017/18, 2018/19 and 2022/23 school years. Shaded areas represent 95% confidence intervals (CI). **B** Cumulative absent days per child per school year, depicted for the three school years, with 95% CI represented as shaded areas around the lines

Figure 3A and B show the illness-related absenteeism rates from 69 primary schools and cumulative absent days over the course of the school season. The mean absenteeism rate varied from 0.9 (95% CI: 0.8–1.1) absent days/100 school days in school year 2017/18 to 2.2 (95% CI: 1.9–2.5) in 2022/23. Substantial differences were observed between schools. A strong seasonal pattern was observed across all years, with peak absenteeism rates between the Christmas and Spring breaks reaching up to 1.7 (95% CI: 1.4–2.0) and 3.2 (95% CI: 2.7–3.9) absent days/100 school days in 2017/18 and 2022/23, respectively (Fig. 3A). These absenteeism rates translate into a cumulative total of 4.7 (95% CI: 4.4–5.1) illness-related absent days per child in 2022/23. Prior to the pandemic, this number was less than half, with 1.8 (95% CI: 1.7-2.0) days during the 2017/18 school year and 2.1 (95% CI: 1.9–2.2) days during the 2018/19 school year (Fig. 3B). Absenteeism rates in 2022/23 in the five schools included in the class-level dataset did not differ significantly from those in the school-level dataset.

### Association between school characteristics and illness-related absenteeism rates

**Fig. 4:**
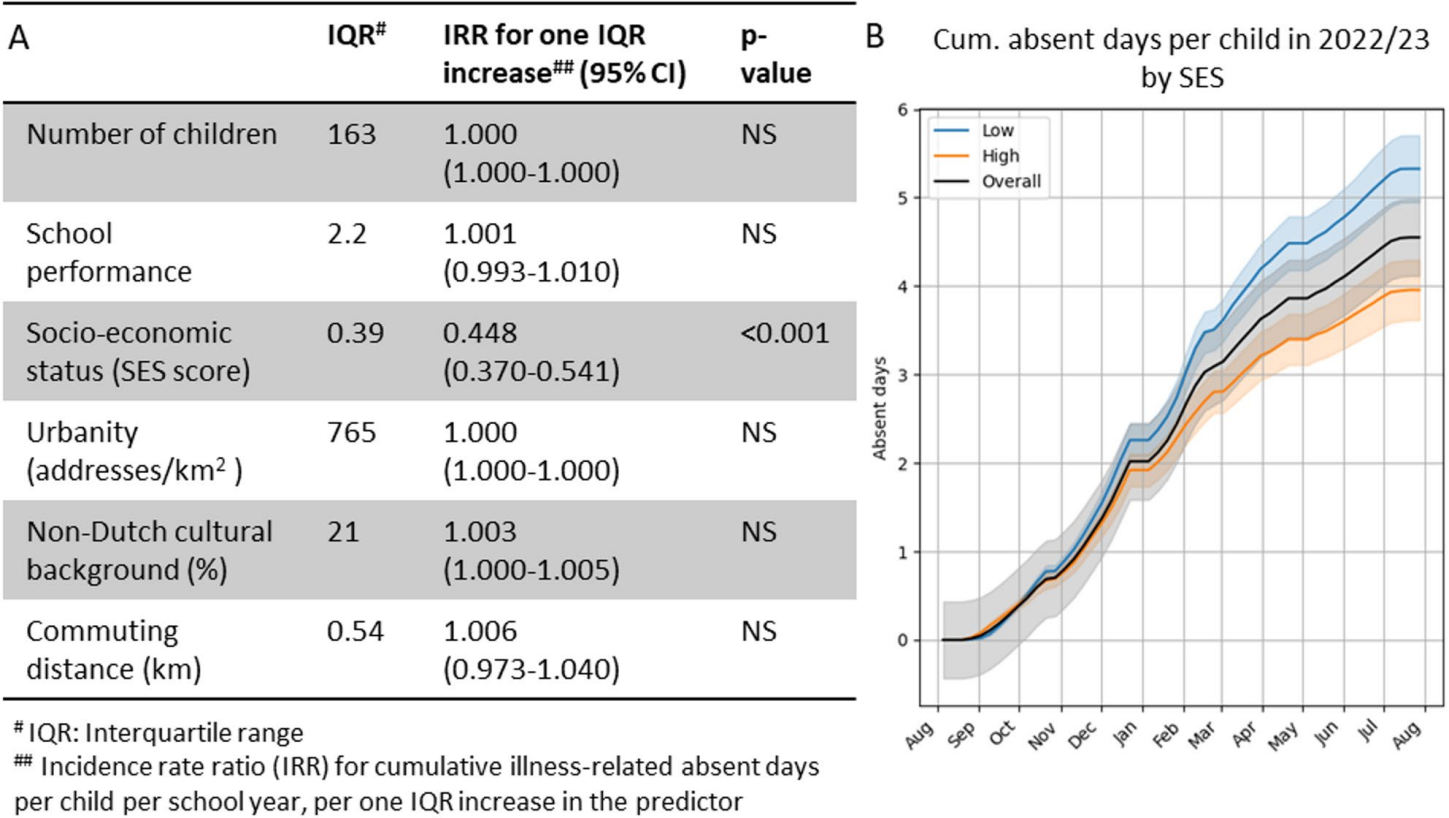
**A** Estimated effects of school characteristics on cumulative illness-related absent days per child per school year, based on a generalized linear mixed model including data from all school years. Results are presented as incidence rate ratios (IRRs) per one interquartile range (IQR) increase in each predictor. **B** Comparison of mean cumulative illness-related absent days per child between schools in the lower and upper halves of SES scores (2022/23 school year shown as example)

Of the investigated school characteristics, only the SES (socioeconomic status) score of the neighbourhood was associated with school absenteeism. A one interquartile range (IQR) increase in the SES score (0.39 points) was associated with a 55.2% decrease in the cumulative number of illness-related absent days per child per school year (Fig. 4A). Figure 4B shows that students in schools in the lower half of SES scores missed roughly 1.5 more days per school year than those in the upper half.

### Correlation between illness-related absenteeism rates and ARI primary care visits

**Fig. 5:**
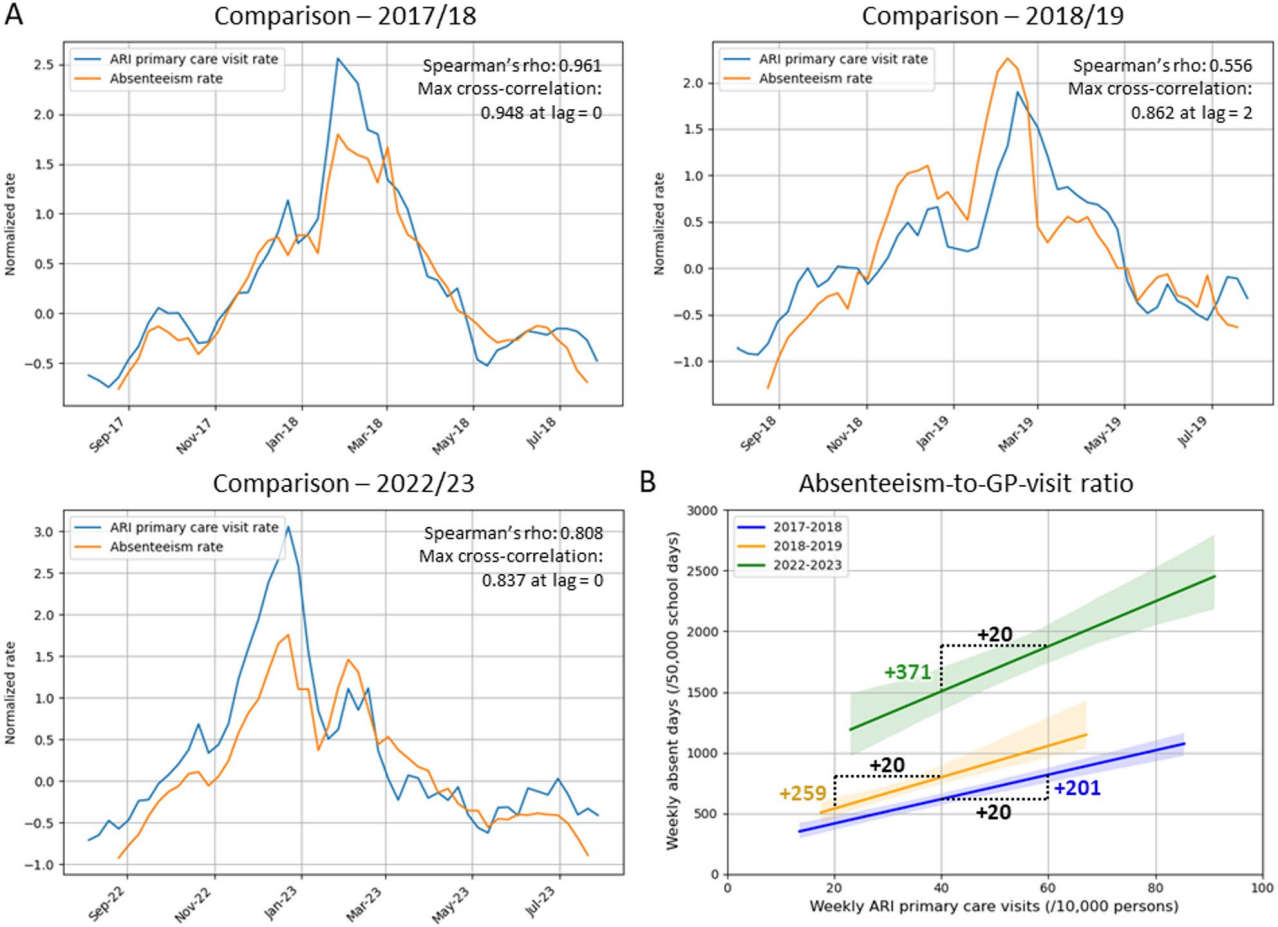
**A** Relationship between normalized illness-related absenteeism rates (weekly absent days/50,000 school days) and ARI primary care visit rates (weekly visits per 10.000 persons), with monotonic correlation assessed using Spearman’s rho and linear correlation measured using cross-correlation. **B** The relationship between the illness-related absenteeism rate and the ARI primary care visit rates during the peak season between the Autumn and Spring break, with linear regression trendlines (including 95% confidence intervals) fitted for the 2017/18, 2018/19, and 2022/23 school years. The slopes represent the absenteeism-to-GP-visits ratio, with the expected increase in absent days for a 20-unit increase in GP visit rates annotated

Figure 5 A shows the relationship between weekly illness-related absenteeism rates in primary school-aged children (4–12 years) and ARI-related primary care visits (5-9-year age group) during the 2017/18, 2018/19, and 2022/23 school years. In each year, ARI primary care visits peaked during the winter months, similar to illness-related absenteeism rates. A strong monotonic correlation was observed between both rates (Spearman’s rho ranging from 0.566 to 0.961, all p-values < 0.01); when the absenteeism rate increased, the GP visit rate also increased, and vice versa. In addition, a strong linear correlation was found in all years, with maximum cross-correlation coefficients of 0.948 at lag 0 in 2017/18, 0.862 at lag 2 in 2018/19, and 0.837 at lag 0 in 2022/23 (all p-values < 0.001), indicating proportional changes in absenteeism and GP visit rates. In 2018/19, a two-week lag was observed, with peaks in school absenteeism preceding increases in ARI-related primary care consultations.

In addition to the observed differences in absolute absenteeism rates between pre- and post-COVID school years, the absenteeism-to-GP-visits ratio also varied across years, as shown in Fig. 5B. For instance, when we focus on the peak season (Autumn to Spring break), an increase of 20 in the weekly ARI primary care visits (/10,000 persons) was associated with an increase of 201, 259 and 371 in absent days/50,000 school days for the 2017/18, 2018/19, and 2022/23 school years, respectively.

### Illness-related vs. all authorized absences

**Fig. 6:**
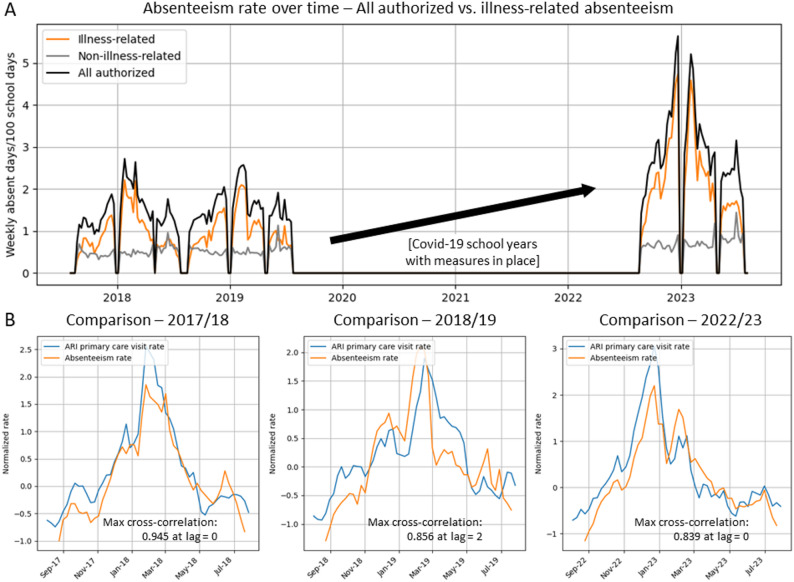
**A** Authorized absenteeism vs. the subcategory illness-related absenteeism during the 2017/18, 2018/19 and 2022/23 school years. **B** Relationship between normalized authorized absenteeism rates (absent days/100 school days) and ARI primary care visit rates (weekly visits per 10.000 persons), with monotonic correlation assessed using Spearman’s rho and linear correlation measured using cross-correlation

In sensitivity analysis, we used authorized absences of any cause instead of illness-related absences. Illness-related absences accounted for 70.9% of all authorized absences. Authorized absences of any cause showed a similar temporal pattern. This is explained by the fact that non-illness-related authorized absences did not exhibit a clear seasonal trend and therefore did not substantially affect the overall pattern (Fig. 6A). Consequently, the correlation between school-level absenteeism rates and ARI primary care visit rates remained comparable to that observed for illness-related absenteeism (Fig. 6B).

## Discussion

This study evaluated the potential of aggregated primary school illness-related absenteeism data for syndromic ARI surveillance by analysing school and healthcare datasets. Our survey among parents of absent children indicated that approximately two-thirds of absences were attributed to ARI. The absences showed clear spatiotemporal clustering within and between schools and absenteeism rates exhibited a clear seasonal pattern, peaking between the Autumn and Spring breaks. Furthermore, absenteeism patterns were strongly correlated with ARI primary care visit rates for the same age-group. Together, these findings support the use of absenteeism rates to monitor ARI rates in Dutch primary school children. Our study also identified other factors influencing school absenteeism: rates were significantly higher in schools located in neighbourhoods with lower SES-scores, and in school-year 2022/23, rates more than doubled compared to pre-pandemic levels.

Our survey among parents of children absent due to illness, conducted over a seven-week period in late autumn and early winter, indicated that approximately two-thirds of absences were attributed to ARI, aligning with evidence that ARI is the leading cause of illness in primary school-aged children [17]. However, absenteeism rates do not perfectly reflect ARI prevalence, as some absences result from GI or other causes, including chronic respiratory conditions, mental health issues, or parental decisions to keep children home for non-medical reasons [18–20]. Also, because reasons are self-reported by parents, they may differ from how schools or health professionals would classify the same episode. Clustering of absenteeism cases within schools, particularly within classes, suggests in-school transmission or a common source of infection, consistent with established transmission dynamics where close contact and high interaction rates facilitate disease spread [21, 22]. Our survey results further support this, indicating that a significant proportion of ARI cases likely originate outside the household, potentially within schools. Exploring this spatiotemporal clustering may aid in distinguishing school-acquired from community-introduced infections and inform the contribution of schools to broader transmission dynamics, but warrants further investigation into appropriate statistical methods for quantitative analysis.

Illness-related absenteeism in primary schools followed a clear seasonal pattern, peaking between the Autumn and Spring breaks. The absenteeism rates observed in this study align with those reported in other European countries [8, 23], though differences in study periods and definitions limit direct comparisons. Absenteeism increased notably in the post-pandemic school year compared to pre-pandemic years, possibly due to a rebound effect, where reduced exposure to respiratory infections during the pandemic heightened susceptibility once restrictions were lifted [24, 25], or shifts in health behaviours [26]. Follow-up studies are needed to determine whether this increase is temporary or reflects longer-term changes. Additionally, absenteeism rates were significantly higher in schools located in lower SES neighbourhoods, consistent with research linking lower SES to increased health vulnerabilities [27]. At the same time, this SES gradient does not necessarily only reflect differences in underlying infection rates; SES may also influence behavioral factors that shape whether children stay home when mildly unwell. Nevertheless, this is an important finding, as children in lower SES contexts already often face greater challenges in education, and higher illness-related absence may add to these existing disadvantages.

Illness-related absenteeism rates (children aged 4–12) strongly correlated with ARI primary care visit rates (5-9-year age group), despite the age groups not fully overlapping. Compared to a systematic review that reported a moderate correlation between school absenteeism and influenza-like illness (ILI) incidence in community surveillance, our findings are at the higher end of the observed range [12]. Despite this strong correlation, absenteeism and GP visit rates captures different aspects of disease burden. Absenteeism reflects a broader range of circulating infections, while the primary care data include only ARI-related consultations. Moreover, absenteeism encompasses a wide spectrum of illness severity, whereas GP visits primarily represent more severe cases. Our absenteeism-to-GP visits ratio suggests that, on a weekly basis, substantially more children are absent from school due to illness than visit a GP for ARI, with estimates in the range of 10-20x. A similar patterns has been observed in community-based studies. In a UK cohort study of childhood respiratory infections, only 8.1% of respiratory tract infection episodes resulted in a GP consultation [4]. This indicates that, as in our data, only a small proportion of childhood respiratory illnesses lead to healthcare use. Although the correlation remained strong across all years, the absenteeism-to-GP visit ratio varied, likely reflecting annual differences in ARI severity that influence both school absence and healthcare-seeking behaviour [28]. Variability in the proportion of absences due to non-ARI illnesses may further contribute to these fluctuations.

In addition to the strong correlation observed between illness-related absenteeism and ARI-related primary care visits, we found that analyses based on authorized absences of any cause yielded comparable results. This likely reflects that illness-related absences comprised the majority of authorized absences and largely shaped their temporal pattern, while other authorized absences (such as family events) did not show a consistent seasonal trend. Previous studies have shown that increasing specificity, from all-cause to illness-specific absenteeism, generally strengthens the correlation with syndromic surveillance indicators [11, 12]. The finding that authorized absenteeism showed a similar seasonal profile indicates that, for surveillance purposes, authorized absenteeism may offer sufficient specificity, thereby eliminating the need to collect and process specific reasons for absence. This increases the generalizability of our findings to contexts where only total authorized absences are available.

This study has several strengths. First, it integrates parental surveys on reasons for absenteeism, illness-related school absenteeism data, and ARI-related primary care visit rates from the same age group, providing a comprehensive assessment of the potential of absenteeism data for ARI surveillance in primary school children. Second, having class-level absenteeism data from five schools, rather than only aggregated school-level data, enabled a more detailed analysis of clustering patterns, possibly reflecting in-school transmission. Additionally, by incorporating school characteristics, the study accounted for contextual factors influencing absenteeism rates, while its coverage of both pre- and post-pandemic school years provided insights into long-term absenteeism trends and demonstrated the sustained correlation with primary care visit rates over time. Finally, absenteeism data from 69 schools were passively collected, making it a scalable and cost-effective approach for surveillance. Since school absenteeism data are collected daily, with appropriate technical integration across school administration systems and the required agreements for data sharing, they could support a near real-time surveillance system, with school-level aggregation currently being the most feasible level for routine implementation due to variation in class-level reporting practices. If sufficiently sensitive, surveillance based on absenteeism data would offer several potential public-health applications. First, it could improve estimates of infection burden in children by capturing mild illness episodes that do not result in GP consultations, thereby informing public-health policy such as vaccination strategies. Second, it could allow earlier identification of school outbreaks by municipal health services, enabling timely school-level responses. Third, it could support evaluation of school-based interventions such as ventilation adjustments, hygiene programs or vaccination strategies, by providing a routinely collected outcome measure. Finally, it could provide early warning signals for broader community trends, as increases in school absenteeism may precede rises in ARI-related healthcare utilization. Although respiratory infections make up the largest share of childhood illness, absenteeism is expected to also reflect noticeable spikes caused by other pathogens, including highly infectious diseases such as measles or an emerging pandemic pathogen.

Despite its potential for syndromic surveillance, absenteeism data have inherent limitations. First, absenteeism rates do not perfectly reflect ARI. Robust validation would require microbiological confirmation from the same school population and over the same period as the absenteeism records. Secondly, our survey data covered only seven weeks, limiting insight into how absenteeism reasons may vary over time. For example other seasonal conditions, such as hay fever, and the changing distribution between ARI and GI were not fully captured. A third limitation is the inherent exclusion of weekends and holidays. This restricts the use of absenteeism data for continuous, year-round community surveillance. While missing holiday data were interpolated, the impact of school closures on disease transmission was not accounted for. Future research could explore this effect, as it may provide valuable insights into the role of school closures in mitigating disease spread. Finally, the study was limited to a subset of Dutch primary schools, located mainly in more urban areas, which may affect generalizability of the results. Because these schools were selected based on data availability and quality, they likely had more consistent absenteeism recording practices then the broader school population. Variation in recording practices across schools remains an important consideration for scalability.

## Conclusion

In conclusion, this study validated that school absenteeism data can be used for ARI surveillance in primary school children. As an accurate, low-cost, and non-invasive data source, absenteeism records offer a valuable alternative and complement to more time-consuming and intrusive surveillance methods.

## Supplementary Information

Supplementary Material 1.

## Data Availability

The class-level and school-level absenteeism data and GP-based surveillance data analyzed in this study were obtained from third-party data providers for the specific purposes of this project. The authors do not have permission to share these data. Access requests should therefore be directed to the respective data providers: absenteeism data via The Implementation Group, and GP-based surveillance data via NIVEL. The survey dataset generated and analyzed during the current study is available from the corresponding author (P.C.J.L. Bruijning-Verhagen,p.bruijning@umcutrecht.nl) upon reasonable request.

## Abbreviations

ARI: Acute respiratory illness
BRIN: Unique school identifier (in Dutch:‘Basisregistratie Instellingen’
DUO: Dutch Education Executive Agency
GI: Gastrointestinal illness
GLMM: Generalized linear mixed-effects model
GP: General practitioner
ICPC: International classification of primary care
IQR: Interquartile range
IRR: Incidence rate ratio
Nivel: The Netherlands Institute for health services research
SES: Socioeconomic status
