# Supplementary material for: School absenteeism data for surveillance purposes: a proxy for acute respiratory infection rates
Source: BMC Public Health. 2026 Mar 17;26:1346. doi: 10.1186/s12889-026-27014-y (PMC13107663; doi:10.1186/s12889-026-27014-y)
Supplement: Supplementary file 1 — Supplementary Material 1. [file 12889_2026_27014_MOESM1_ESM.pdf]

SUPPLEMENTARY INFORMATION

Title: School absenteeism data for surveillance purposes: a proxy for acute respiratory infection rates

Authors: C.M. de Korne[1], M. Hooiveld[2], A.J. van Hoek[3], P.C.J.L Bruijning-Verhagen[1]

Affiliations:
[1]*Julius Center for Health Sciences and Primary Care, UMC Utrecht, Utrecht, The Netherlands.*
[2]*Nivel, Utrecht, the Netherlands.*
[3]*Centre for Infectious Disease Control, National Institute for Public Health and the Environment, Bilthoven, The Netherlands.*

Correspondence: P.C.J.L Bruijning-Verhagen, p.bruijning@umcutrecht.nl

---

Supplement 1. Categorization of survey-reported symptoms into ARI and GI

During a seven-week period (Nov-Dec 2023), an illness survey was conducted in five primary schools. Parents of absent students were invited to report the reason for absence and symptoms via an anonymous online form. Each response included:

- One main reason for absence, selected from:
    - Cold/flu
    - Fatigue
    - Accident/injury
    - Abdominal pain/bowel complaints
    - Other
- One or more symptoms, selected from:
    - Fever
    - Coughing
    - Runny nose
    - Sore throat
    - Shortness of breath
    - Earache
    - Muscle pain
    - Headache
    - Vomiting and/or diarrhoea
    - None of the above

Based on the combination of the selected reason and symptoms, each case was classified into one of three categories: acute respiratory infection (ARI), gastrointestinal illness (GI), or other/unknown, using the following rule-based approach:

1. Primary ARI classification

   An absence was classified as ARI if:
    - The reason was cold/flu, fatigue, other, or left blank, and the symptoms included coughing and/or runny nose; or
    - The reason was cold/flu and the symptom sore throat was reported.
2. GI classification

   An absence was classified as GI if:
    - The reason was abdominal pain/bowel complaints; or
    - The symptoms included vomiting and/or diarrhoea.
3. Fallback ARI classification

   If the criteria above were not met, the case was still classified as ARI if:

- The reason was cold/flu; or
- The symptoms included sore throat, or earache.

4. Other/Unknown

Cases that did not meet any of the above conditions were classified as other/unknown.

Parents were also asked whether other household members had experienced similar symptoms. Response options included: (1) symptoms started earlier in household members, (2) symptoms started later in household members, or (3) no other household members had similar symptoms. Based on their responses, each episode was classified as either ‘prior household symptoms’ (symptoms had started earlier in at least one other household member) or ‘no prior household symptoms’ (suggesting the child was the index case who likely contracted the illness outside the household, possibly at school).

Supplement 2. School characteristics and representativeness

69 primary school meeting the following criteria were included in the school-level absenteeism dataset:

- Availability of absenteeism data for the full school years 2017/18, 2018/19 and 2022/23.
- Sufficient data quality, defined as coverage of more than 70% of expected school days.
- Classification as regular primary schools, while special primary education and special schools were excluded to ensure the dataset reflects absenteeism patterns in mainstream educational environments.

The 69 primary schools were all located within the municipal health service regions (in Dutch: GGD regio) of Amsterdam, Hollands-Midden, and Limburg (Figure S1A). The absenteeism dataset from these schools was enriched by linking it via BRIN to detailed school information and educational data provided by The Dutch Education Executive Agency (in Dutch abbreviated as ‘DUO’). DUO provides open-access information such as total number of children enrolled in each school, percentage of children with a non-Dutch background, median distance travelled by children to reach the school, and school performance based on standardized test results of children completing primary school. Each school was also assigned to its respective holiday region (North, Central, South), a regional classification used for scheduling school holidays (Table S1). Additionally, the degree of urbanity (number of addresses per square kilometre) and the socio-economic status (SES) score of the neighbourhood were included as school characteristics. Because individual-level data were not available and most children in our dataset lived close to their school (median 0.73 km), neighborhood characteristics provided the best available proxies for these characteristics. These data were obtained from Statistics Netherlands (in Dutch abbreviated as CBS) (Table S1). All school characteristics were robustly scaled by subtracting the median and dividing by the interquartile range, ensuring comparability across predictors and reducing sensitivity to outliers.

Unlike in some other countries, seasonal influenza vaccination is not part of the routine childhood program in the Netherlands. Vaccination coverage is therefore very low, which is why it was not included as a school characteristic in this study.

Table S1 Open data from DUO and CBS

Source	Dataset	URL
DUO	Primary school locations (*Schoolvestigingen basisonderwijs*)	https://duo.nl/open_onderwijsdata/primair-onderwijs/scholen-en-adressen/schoolvestigingen-basisonderwijs.jsp
DUO	Student numbers (*Historisch overzicht leerlingenaantallen primair onderwijs*)	https://duo.nl/open_onderwijsdata/primair-onderwijs/aantal-leerlingen/historisch-overzicht-leerlingen-schoolvestiging.jsp
DUO	Non-native students numbers (*Aantal NOAT-leerlingen per schoolvestiging*)	https://duo.nl/open_onderwijsdata/primair-onderwijs/aantal-leerlingen/noat-leerlingen-schoolvestiging.jsp

DUO	Travel distance (*Gemiddelde afstand tussen woonadres en school*)	https://duo.nl/open_onderwijsdata/primair-onderwijs/aantal-leerlingen/gemiddelde-afstand-woonadres-school.jsp
DUO	Final test scores (*Gemiddelde eindscores*)	https://duo.nl/open_onderwijsdata/primair-onderwijs/aantal-leerlingen/gemiddelde-eindscores.jsp
CBS	Urbanity (*Kerncijfers wijken en buurten 2004-2024*)	https://www.cbs.nl/nl-nl/reeksen/publicatie/kerncijfers-wijken-en-buurten
CBS	Socio-economic status (*1. SES per postcode, 2014-2019, excl. studenten; 2. Sociaal-economische status per postcode, 2020 en 2021, excl. studenten*)	1. https://www.cbs.nl/nl-nl/maatwerk/2022/40/ses-per-postcode-2014-2019-excl-studenten. 2. https://www.cbs.nl/nl-nl/maatwerk/2023/33/sociaal-economische-status-per-postcode-2020-en-2021

The school characteristics of the schools were compared to those of all primary schools in the Netherlands to assess representativeness. Distributions of these characteristics were summarized using the median and interquartile range (IQR) and compared using the Mann-Whitney U test to assess significant differences. On average, the number of children per school was higher than the national average for Dutch primary schools (median: 281 vs 200, IQR: 212-378). The schools also had higher percentages of children with non-Dutch cultural backgrounds (median: 13% vs 6%, IQR: 7-28%) and were more frequently located in urban municipalities (median addresses per km^2: 1835 vs 1245, IQR: 1464-2248) with a lower socio-economic status (median SES score: -0.04 vs 0.09, IQR: -0.24-0.13). School performance of the schools in this study, based on end-of-primary-school assessment scores, was comparable to the national average for Dutch primary schools (Figure S1B).

A

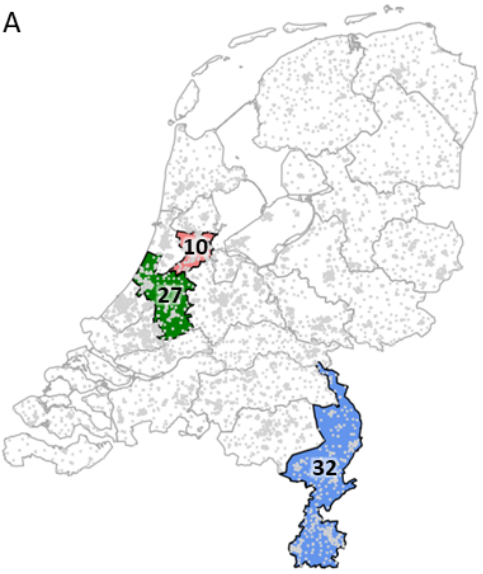

GGD regions: Amsterdam, Hollands-Midden, Noord- en Zuid-Limburg

B

	Study schools#	All Dutch schools#	p-value##
Number of children	281 (212-378)	200 (126-291)	***
School performance###	0.15 (-1.01-1.13)	0.01 (-0.98-0.86)	NS
Socio-economic status (SES score)	-0.04 (-0.24-0.13)	0.09 (-0.07-0.20)	***
Urbanity (addresses/km^2)	1835 (1464-2248)	1245 (598-1969)	***
Non-Dutch cultural background (%)	13 (7-28)	6 (2-16)	***
Travel distance (km)	0.73 (0.53-1.04)	0.67 (0.38-1.14)	NS

Values are shown as median (IQR)
<0.05: *, <0.01: **, <0.001: ***, Mann-Whitney U test
Based on end-of-primary-school assessments scores

Figure S1 A) Overview of the number of schools included in the school-level absenteeism dataset from the three different GGD regions; 69 in total. The grey dots represents all Dutch primary schools. B) Baseline characteristics of the 69 schools compared to all Dutch schools.

Supplement 3. Definitions of illness-related absenteeism

In Dutch schools, absences are classified as either authorized or unauthorized, with authorized absences covering illness and exceptions such as family events. For each absence, a reason is always provided by the school as a free-text entry. Absences were classified as illness-related if they were marked as authorized and contained the Dutch word for illness (*ziek*), excluding cases mentioning the Dutch word for hospital (*ziekenhuis*). Table S2 provides an overview of the ten most common reasons classified as illness-related, along with their frequency.

Table S2 Free-text reasons classified as illness-related

Reason	Translation	Frequency
Ziek	Sick	179584
Na informeren ziek	Reported sick after school contact	150667
Ziek (Z)	Sick (S)	30611
Ziek (ZK)	Sick (SK)	3591
afwezig ziek na informeren	Absent sick after school contact	2640
Ziek Dag	Sick day	2264
M Ziek	M sick	2199
Ziekte na informeren	Illness reported after school contact	1394
Ziekte	Illness	708
Ziek 's Morgens	Sick morning	450
All other reasons containing 'ziek'		1617

Supplement 4. GP practices part of the Nivel Primary Care Database

This study used data from the Nivel Primary Care Database, which collects routinely recorded electronic health records from a nationwide network of general practices in the Netherlands. For this analysis, data was included from over 400 GP practices. These practices are geographically distributed across all GGD regions. Figure S2 shows the spatial distribution of the contributing practices, with GGD regions coloured by the number of participating practices.

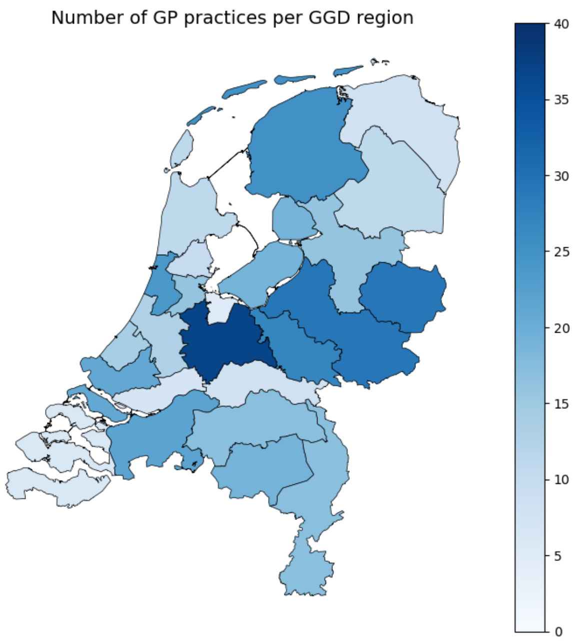

Figure S2 Geographic distribution of the >400 GP practices contributing to the Nivel Primary Care Database. GGD regions are colored according to the number of included practices.
